# Clinical significance of Girdin expression detected by immunohistochemistry in non-small cell lung cancer

**DOI:** 10.3892/ol.2013.1745

**Published:** 2013-12-09

**Authors:** JING-YING SONG, PING JIANG, NING LI, FENG-HUA WANG, JUN LUO

**Affiliations:** 1 Department of Pathology, The 309th Hospital of Chinese People’s Liberation Army, Beijing 100091, P.R. China; 2 The Key Laboratory of Geriatrics, Beijing Hospital and Beijing Institute of Geriatrics, Ministry of Health, Beijing 100730, P.R. China

**Keywords:** Girdin, non-small cell lung cancer, Ki-67, metastasis

## Abstract

Girdin protein has been implicated in cell migration and proliferation control. Previous evidence has confirmed that Girdin is a pivotal protein during cancer progression. To date, no evidence has been identified for the clinical significance of Girdin expression in non-small cell lung cancer (NSCLC). The current study aimed to investigate the expression and clinical significance of Girdin protein in NSCLC. In total, 36 tumor samples were obtained from patients undergoing surgery for NSCLC at The 309th Hospital of Chinese People’s Liberation Army (Beijing, China). The protein expression of Girdin was determined by immunohistochemistry analysis and the levels of Girdin protein were significantly higher in tumor samples than in distal normal lung tissue. A significant correlation was identified between Girdin overexpression and blood vessel infiltration of the tumor (P=0.013). Furthermore, analysis found that the Girdin-high phenotype was not associated with higher Ki-67 score. Girdin protein was frequently overexpressed in NSCLC and expression of Girdin was associated with blood vessel infiltration. The results of the present study suggest that Girdin should be considered as a potential marker for the prognosis of NSCLC; however, future studies are required to confirm theses results.

## Introduction

Lung cancer is the most common cause of cancer-related mortality for males and females worldwide (1). Non-small cell lung cancer (NSCLC) accounts for ~85% of all cases of lung cancer with an overall 5-year survival rate of <20.0%, as the majority of patients are diagnosed at a late stage and are unsuitable for curative surgery (2). Squamous cell carcinoma (SCC) and adenocarcinoma (AC) represent the majority of NSCLCs.

According to a number of previous randomized clinical trials, adjuvant chemotherapy is now considered to be the unequivocal standard treatment for NSCLC patients. However, only a proportion of patients benefit from adjuvant chemotherapy, while others may succumb to metastasis derived from the malignancy (3). Thus, it is imperative to identify novel prognostic biomarkers that precisely predict metastasis in patients with NSCLC. Such advances are likely to be useful to stratify patients with NSCLC and select high-risk patients who should receive aggressive adjuvant chemotherapy.

Girdin is overexpressed in various solid tumors, including breast cancer, cervical carcinoma, lung, thyroid (4) and colorectal (5) cancer, and glioblastoma (6). The present study, consistent with previous studies, found that the expression of Girdin correlates with tumor metastasis and may be a potential new distant metastasis biomarker of breast and colorectal cancer (4,5,7–10). Girdin locates at the crossroad of G protein and tyrosine kinase receptor signaling (11), and promotes cell migration via recruiting and controlling the actin filaments (4,12). In addition, Girdin facilitates cell proliferation by activating the mitogenic signals (13). Increasing evidence has confirmed that Girdin is necessary for cell migration and proliferation, as well as tumor metastasis and angiogenesis (4,13–15). However, the correlation between Girdin expression and clinical features in NSCLC remain unclear.

The aim of the present study was to assess the expression of Girdin in a cohort of 36 consecutive patients with NSCLC and correlate its expression with survival and other clinicopathological parameters.

## Materials and methods

### Ethics statements

All experiments were performed in strict accordance with the recommendations in the Guide for the Care and Use of Laboratory Animals of the National Institutes of Health. The protocol was approved by the Animal Care and Use Committee of The 309th Hospital of Chinese People’s Liberation Army (Beijing, China).

### Biopsy specimens

Paraffin-embedded sections of 36 NSCLCs were obtained from the Department of Pathology of The 309th Hospital of Chinese People’s Liberation Army, together with regional lymph node dissection, between January 2010 and December 2012. Patients who had received preoperative chemotherapy or radiotherapy were excluded. The study was approved by the ethical committee of The 309th Hospital of Chinese People’s Liberation Army prior to initiation.

### Immunohistochemistry

All samples were fixed in 10% buffered formalin and embedded in paraffin, and tissue sections (4 μm thick) were obtained. All sections were deparaffinized and dehydrated with graded alcohol. The sections were then washed for 10 min in phosphate-buffered saline (PBS; pH 7.2). The endogenous peroxidase activity was quenched by incubation in methanol containing 3% H_2_O_2_ for 10 min at room temperature, then heated for 30 min at 95°C to repair antigens and finally rinsed in PBS. Following several washes in PBS, the sections were blocked with goat serum for 15 min at room temperature and then incubated with rabbit polyclonal Girdin primary antibody (Santa Cruz Biotechnology, Inc., Santa Cruz, CA, USA) overnight at 4°C in a humidified chamber. In negative control sections, the primary antibody was replaced by PBS. All slides were treated with polymer enhancer (Reagent A; Zhongshan Jinqiao Biotechnology Co., Ltd., Beijing, China) for 20 min at room temperature. Following a complete wash in PBS, the slides were treated with goat anti-rabbit antibody (Reagent B; Zhongshan Jinqiao Biotechnology Co., Ltd.) for 30 min at room temperature. Following an additional complete wash in PBS, the slides were developed in freshly prepared diaminobenzidine solution for 8 min and then counterstained with hematoxylin, dehydrated, air-dried and mounted.

### Evaluation of score

Slides were reviewed independently by two pathologists to evaluate the staining pattern of the protein separately under the light microscope (Eclipse 80i, Nikon, Tokyo, Japan). In scoring the expression of Girdin protein, the extent and intensity of immunopositivity were considered. The intensity of positivity was scored as follows: 0, negative; 1, weak; 2, moderate; and 3, strong. The extent of positivity was scored according to the percentage of cells showing positive staining as follows: 1, <10%; 2, 11–50%; 3, 51–75%; and 4, >75%. The final score was determined by multiplying the intensity and extent of positivity scores, yielding a range between 0 and 12. The expression for Girdin was considered positive when the scores were >1. For the evaluation of immunoreactivity of Ki-67, 200 cells from five representative fields of each section were randomly selected and counted in a blinded manner by two independent observers. Inconsistent data were discussed by the observers until final agreements were reached. The expression positivity was graded and counted as follows: Low, <25%; moderate, 25–50%; and high, >50%.

### Statistical analysis

All data were analyzed using SPSS 13.0 software (SPSS, Inc., Chicago, IL, USA). The correlation between Girdin and other parameters was investigated using the χ^2^ test, Spearman’s rank correlation or an independent t-test when appropriate. Linear regression was used to evaluate the correlation among Girdin, Ki-67 and other parameters. P<0.05 was considered to indicate a statistically significant difference.

## Results

### Clinical data

Specimens were obtained from archived paraffin-embedded tissue sections of 36 patients with NSCLC. In the cohort of NSCLC patients, 26 were male and 10 were female, with a median age of 58 years (range, 31–77 years). According to the World Health Organization classification of lung tumors published in 2001 (16), NSCLC patients were classified as follows: AC, 21 cases; SCC, 12 cases; other types, three cases; well- and moderately differentiated carcinoma, 24 cases; and poorly differentiated carcinoma, 12 cases. In the 36 NSCLC cases, 23 exhibited lymph node metastasis and 11 exhibited blood vessel infiltration.

### Expression of Girdin and Ki-67 in NSCLC

**Figure 1:**
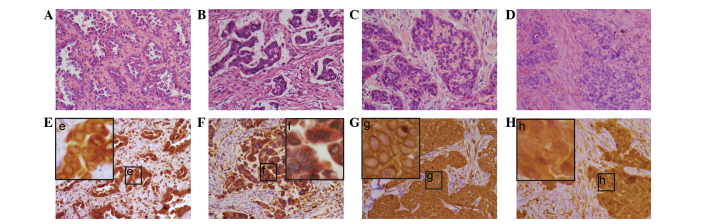
Immunohistochemical staining of Girdin protein in non-small cell lung cancer tissue samples. Tissue sections were immunohistochemically stained with an anti-Girdin antibody. Positive Girdin immunostaining was mainly localized in (F and H) the cytoplasm or (E and G) surrounding the nuclei membrane of (C, D, G and H) squamous cell carcinoma and (A, B, E and F) adenocarcinoma cells (magnification, ×200).

**Figure 2:**
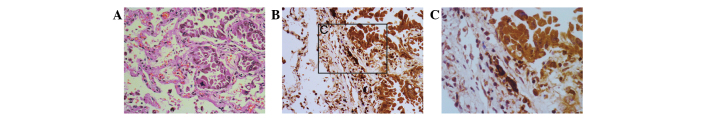
Girdin is present in human lung cancer tissues. (A) Human lung cancer and distal normal tissues were identified by hematoxylin and eosin staining. (B) Protein expression of Girdin was mainly evident in the cytoplasm with yellow or brown-yellow staining. The cancer tissues were strongly stained by anti-Girdin antibody, but staining was less prominent in the distal normal lung tissue. (C) Magnified image of box in B. Magnification, ×200 (A and B) and ×400 (C).

In total, 26 (72.2%) of the 36 NSCLC cases showed positive expression of Girdin. The expression of Girdin in NSCLC tissues was predominantly observed in the cytoplasm or around the nuclei membranes in SCC and AC (Fig. 1A–D). The perinucleus was characterized by thick, rounded, densely stained material around the nucleus (Fig. 1; the lower panel). In total, 11 (30.6%) of the 36 cases exhibited the perinuclear pattern. A significant difference was identified between the NSCLC and distal normal lung tissue (Fig. 2), and eight (22.2%) of the 36 cases showed high expression of Ki-67.

### Correlation between the protein expression of Girdin and clinicopathological parameters in NSCLC

Furthermore, correlation analysis demonstrated that the expression of Girdin was found to significantly correlate with blood vessel infiltration in NSCLC patients. High levels of Girdin were found to correlate with significant blood vessel infiltration (P=0.013). However, no correlations were observed between Girdin expression patterns and the other clinicopathological parameters studied (P>0.1; Table I).

In addition, the correlation between the perinuclear expression pattern of Girdin and the clinicopathological parameters was analyzed, but no significant difference was identified (all P>0.1; Table II).

### Combined Ki-67 and Girdin analysis

The potential correlation between the protein expression of Girdin and Ki-67 in NSCLC was evaluated. Spearman’s rank correlation analysis showed that the expression of Girdin did not correlate with Ki-67 expression in the NSCLC cohort (r=0.125; P=0.468; Table III).

## Discussion

Previously, increasing evidence has shown that Girdin expression is associated with tumor invasion/metastasis, angiogenesis and growth in patients with colorectal and breast cancer (4–13). In the present study, high expression of Girdin protein was detected in 72.2% of NSCLCs, while the expression level of Girdin in the normal lung tissues was extremely low. Furthermore, statistical analysis showed that the expression of Girdin was found to closely correlate with NSCLC differentiation degree and blood vessel infiltration. The positive expression of Girdin was more frequently observed in poorly differentiated cancer and tumors with blood vessel infiltration.

Recently, Natsume *et al* reported that Girdin is required for glioblastoma-initiating stem cells to sustain the stemness and invasive properties. Stable Girdin knockdown in isolated glioblastoma stem cells induced multilineage neural differentiation (6). According to this study, Girdin is pivotal for tumor cell differentiation in NSCLC. Previous studies have suggested that Girdin produces a marked effect by regulating the cytoskeleton of tumor cells (4). Following binding to the actin filaments, Girdin directly controls the migration and invasion of breast cancer cells. In addition, Ohara *et al* found that Girdin interacts with Par-3, a scaffolding protein that is a component of the Par protein complex that has an established role in determining cell polarity (17). Thus, Girdin facilitates tumor metastasis by regulating the cytoskeleton. These results suggested that the increased expression of Girdin may facilitate the development and/or progression of NSCLC.

In the present study, the Spearman’s rank correlation analysis showed no correlation between the protein expression of Girdin and Ki-67. However, previous studies have revealed that Girdin is involved in tumor growth by upregulating a variety of kinases, such as ERK 1/2, Src and STAT5 (13). Therefore, when the expression level of Girdin is higher, it promotes tumor growth through these molecules. The results of the present study indicated no correlation between Girdin expression and tumor cell proliferation in the tissues. It appears that more cases are required to investigate the effect of Girdin expression on cancer cell proliferation.

The current study confirmed that Girdin is highly expressed in NSCLC. This result is consistent with previous studies, which have reported that Girdin is highly expressed in breast and colorectal cancer (4,5,7–10). It has been documented that Girdin promotes cell proliferation and migration (13). In addition, increasing evidence has confirmed that high expression of Girdin is associated with tumor metastasis and poorer postoperative, disease-specific survival (5,9,10). Based collectively on the aforementioned results, we proposed that Girdin may also be involved in the tumorigenesis/progression of NSCLC.

In conclusion, the present study is the first to demonstrate that overexpression of Girdin closely correlates with the malignant progression in patients with NSCLC. Girdin expression may have clinical value as a new target for the treatment of lung cancer. Future studies with larger cohorts of patients are required to confirm the results of the current study and to establish a prognostic role for this protein.

## Figures and Tables

**Table I:** Characteristics of 36 patients with NSCLC and the correlation between Girdin protein expression and clinicopathological variables.

		Girdin protein expression		
			
Variables	Total (n=36), n	Low (n=10), n (%)	High (n=26), n (%)	P-value
Age, years				0.644
<60	18	5 (27.8)	13 (72.2)	
≥60	18	5 (27.8)	13 (72.2)	
Gender				0.580
Male	26	7 (26.9)	19 (73.1)	
Female	10	3 (30.0)	7 (70.0)	
Tumor size, cm				0.519
<3.5	16	4 (25.0)	12 (75.0)	
≥3.5	20	6 (30.0)	14 (70.0)	
Histological type				0.575
SCC	12	2 (16.7)	10 (83.3)	
AC	21	7 (33.3)	14 (66.7)	
Others	3	1 (33.3)	2 (66.7)	
Lymphatic infiltration				0.527
Yes	23	6 (26.1)	17 (73.9)	
No	13	4 (30.8)	9 (69.2)	
Blood vessel infiltration				0.013
Yes	11	0 (0.0)	11 (100.0)	
No	25	10 (40.0)	15 (60.0)	
Histological grade				0.330
Well-differentiated	6	3 (50.0)	3 (50.0)	
Moderately differentiated	18	5 (27.8)	13 (72.2)	
Poorly differentiated	12	2 (16.7)	10 (83.3)	

NSCLC, non-small cell lung cancer; SCC, squamous cell carcinoma; AC, adenocarcinoma.

**Table II:** Characteristics of 36 patients with NSCLC and the correlation between Girdin protein expression and clinicopathological variables.

		Girdin expression around nuclei		
			
Variables	Total (n=36), n	Sig. (n=11), n (%)	Not sig. (n=25), n (%)	P-value
Age, years				0.073
<60	18	3 (16.7)	15 (83.3)	
≥60	18	8 (44.4)	10 (55.6)	
Gender				0.335
Male	26	9 (34.5)	17 (65.5)	
Female	10	2 (20.0)	8 (80.0)	
Tumor size, cm				0.391
<3.5	16	4 (25.0)	12 (75.0)	
≥3.5	20	7 (35.0)	13 (65.0)	
Histological type				0.347
SCC	12	5 (41.7)	7 (58.3)	
AC	21	6 (28.6)	15 (71.4)	
Others	3	0 (0.0)	0 (0.0)	
Lymphatic infiltration				0.367
Yes	23	8 (34.5)	15 (65.5)	
No	13	3 (23.1)	10 (76.9)	
Blood vessel infiltration				0.551
Yes	11	3 (27.3)	8 (72.7)	
No	25	8 (32.0)	17 (68.0)	
Histological grade				0.182
Well-differentiated	6	3 (50.0)	3 (50.0)	
Moderately differentiated	18	3 (16.7)	15 (83.3)	
Poorly differentiated	12	5 (41.7)	7 (58.3)	

NSCLC, non-small cell lung cancer; sig., significant; SCC, squamous cell carcinoma; AC, adenocarcinoma.

**Table III:** Correlation between Girdin and Ki-67 protein expression.

Girdin protein expression	Ki-67 (<25%), n (%)	Ki-67 (25–50%), n (%)	Ki-67 (>50%), n (%)
High	5 (71.4)	14 (66.7)	7 (87.5)
Low	2 (28.6)	7 (33.3)	1 (12.5)

## References

[1] Shin HR, Carlos MC, Varghese C (2012). Cancer control in the Asia Pacific region: current status and concerns. Jpn J Clin Oncol.

[2] Jemal A, Bray F, Center MM, Ferlay J, Ward E, Forman D (2011). Global cancer statistics. CA Cancer J Clin.

[3] Bonomi M, Pilotto S, Milella M, Massari F, Cingarlini S, Brunelli M, Chilosi M, Tortora G, Bria E (2011). Adjuvant chemotherapy for resected non-small-cell lung cancer: future perspectives for clinical research. J Exp Clin Cancer Res.

[4] Jiang P, Enomoto A, Jijiwa M, Kato T, Hasegawa T, Ishida M, Sato T, Asai N, Murakumo Y, Takahashi M (2008). An actin-binding protein Girdin regulates the motility of breast cancer cells. Cancer Res.

[5] Garcia-Marcos M, Jung BH, Ear J, Cabrera B, Carethers JM, Ghosh P (2011). Expression of GIV/Girdin, a metastasis-related protein, predicts patient survival in colon cancer. FASEB J.

[6] Natsume A, Kato T, Kinjo S, Enomoto A, Toda H, Shimato S, Ohka F, Motomura K, Kondo Y (2012). Girdin maintains the stemness of glioblastoma stem cells. Oncogene.

[7] Liu C, Zhang Y, Xu H, Zhang R, Li H, Lu P, Jin F (2012). Girdin protein: a new potential distant metastasis predictor of breast cancer. Med Oncol.

[8] Ling Y, Jiang P, Cui SP, Ren YL, Zhu SN, Yang JP, Du J, Zhang Y, Liu JY, Zhang B (2011). Clinical implications for girdin protein expression in breast cancer. Cancer Invest.

[9] Liu C, Xue H, Lu Y, Chi B (2012). Stem cell gene Girdin: a potential early liver metastasis predictor of colorectal cancer. Mol Biol Rep.

[10] Jun BY, Kim SW, Jung CK, Cho YK, Lee IS, Choi MG, Choi KY, Oh ST (2013). Expression of girdin in human colorectal cancer and its association with tumor progression. Dis Colon Rectum.

[11] Ghosh P, Garcia-Marcos M, Farquhar MG (2011). GIV/Girdin is a rheostat that fine-tunes growth factor signals during tumor progression. Cell Adh Migr.

[12] Weng L, Enomoto A, Ishida-Takagishi M, Asai N, Takahashi M (2010). Girding for migratory cues: roles of the Akt substrate Girdin in cancer progression and angiogenesis. Cancer Sci.

[13] Ghosh P, Beas AO, Bornheimer SJ, Garcia-Marcos M, Forry EP, Johannson C, Ear J, Jung BH, Cabrera B, Carethers JM, Farquhar MG (2010). A Gαi-GIV molecular complex binds epidermal growth factor receptor and determines whether cells migrate or proliferate. Mol Biol Cell.

[14] Enomoto A, Murakami H, Asai N, Morone N, Watanabe T, Kawai K, Murakumo Y, Usukura J, Kaibuchi K, Takahashi M (2005). Akt/PKB regulates actin organization and cell motility via Girdin/APE. Dev Cell.

[15] Kitamura T, Asai N, Enomoto A, Maeda K, Kato T, Ishida M, Jiang P, Watanabe T, Usukura J (2008). Regulation of VEGF-mediated angiogenesis by the Akt/PKB substrate Girdin. Nat Cell Biol.

[16] Brambilla E, Travis WD, Colby TV, Corrin B, Shimosato Y (2001). The new World Health Organization classification of lung tumours. Eur Respir J.

[17] Ohara K, Enomoto A, Kato T, Hashimoto T, Isotani-Sakakibara M, Asai N, Ishida-Takagishi M, Weng L, Nakayama M (2012). Involvement of Girdin in the determination of cell polarity during cell migration. PLoS One.

